# Enteric Methane Emission and Nitrogen Excretion of Lactating Cows Fed Soybean-Hulls as Partial Replacement of Corn Grain

**DOI:** 10.3390/ani15243575

**Published:** 2025-12-12

**Authors:** Lucia Maria Buraschi, Florencia Micoli, Rafael Alejandro Palladino, Alejandra Cuatrin, Carolina Calamante, Leandro Demian Guerrero, Diana Wehrendt, Laura Bibiana Gualdrón-Duarte, Maria Paz Tieri, S. Richard O. Williams, Patricia Ricci

**Affiliations:** 1 National Scientific and Technical Research Council (CONICET), Ciudad Autónoma de Buenos Aires 1425, Argentina; luciaburaschi@gmail.com (L.M.B.);; 2 Faculty of Agricultural Sciences, National University of Mar del Plata (UNMdP), Balcarce 7620, Argentina; 3 Faculty of Agriculture Science, IIPAAS, National University of Lomas de Zamora (UNLZ), Lomas de Zamora B1832, Argentina; 4 Faculty of Agronomy, University of Buenos Aires (UBA), Ciudad Autónoma de Buenos Aires 1417, Argentina; 5 Fundación Instituto de la Leche (FIL), Cuidad Autónoma de Buenos Aires 1012, Argentina; 6 National Institute of Agricultural Technology (INTA) Paraná Experimental Station, Paraná B3100AET, Argentina; 7 Institute for Research on Genetic Engineering and Molecular Biology “Dr. Héctor N. Torres” (INGEBI CONICET), Ciudad Autónoma de Buenos Aires C1428ADN, Argentina; 8 Agricultural Production and Sustainable Development Research Institute (IPADS INTA-CONICET), Balcarce 7620, Argentina; lauragualdronduarte@gmail.com; 9 Rafaela Regional Faculty, National Technological University (UTN), Rafaela 2300, Argentina; 10 Agriculture Victoria Research, Ellinbank, VIC 3821, Australia; richard.williams@agriculture.vic.gov.au

**Keywords:** dairy cattle, methane, nitrogen, energy efficiency, soy hulls, corn grain

## Abstract

Including soybean hulls (SH) in ruminant diets increase the carbon circularity of livestock systems. We evaluated whether SH could partially replace corn grain (CG) in the diet of lactating cows (≈7.5% of the total diet). Six Holstein multiparous cows were fed a 50:50 (dry matter basis) corn-silage to concentrate diet for two 25-day periods in three replicated 2 × 2 Latin Squares using linear mixed models. Feed intake, milk yield and composition, N excretion, CH_4_ production, and ruminal microbial profiles were measured. Enteric CH_4_ was measured in respiration chambers. The DMI of cows fed SH (28 kg DM/d) was greater (*p* = 0.034) than those fed CG (26 kg DM/d), but no differences were observed in milk production (*p* = 0.557) or enteric CH_4_ emission intensity (g/kg milk, *p* = 0.387). Enteric CH_4_ emissions tended to be greater in SH-fed cows (484 g/d) than CG-fed cows (456 g/d; *p* = 0.075), and N excretion was significantly greater in SH than CG cows (*p* < 0.001). No mayor shifts in ruminal microbiota or overall ruminal environment were observed. In conclusion, SH can partially replace CG in diets of lactating dairy cows without affecting production, but potential negative environmental impacts should be considered.

## Simple Summary

The dairy industry is under increasing pressure to reduce its environmental footprint, particularly methane emissions. One strategy to improve the sustainability of milk production is to replace traditional feed ingredients with agricultural by-products that would otherwise be wasted. In our study, we evaluated whether partially replacing corn grain with soybean hulls in the diet of lactating cows affects their productivity and environmental impact. Milk production and composition were not affected, but cows offered soybean hulls ate more feed and had lower feed efficiency than those offered corn grain. Although methane emission per kilogram of milk remained unchanged, cows fed soybean hulls tended to produce a greater total amount of methane emitted per cow than those offered corn grain. Nitrogen excretion was also significantly greater in cows offered soybean hulls compared to those offered corn grain. These findings suggest that potential improvements in nutrient circularity of soybean hulls can be jeopardized by a tendency to increase net enteric methane emissions. Further research is needed to assess the full carbon footprint and sustainability at the whole-farm level to determine if increasing the proportion of soybean hulls in dairy cow diets is advantageous.

## 1. Introduction

The livestock industry is currently facing a complex situation, given the challenge of meeting a growing demand for animal products while simultaneously committing to reduce the environmental impact of food production [1]. Feeding ruminants with high-quality by-products from the agroindustry sector seems to be a ‘win-win’ tool to both reduce the environmental impact of ruminant production and increase the nutrient circularity of the agricultural sector.

Soybean hulls (SH) are widely used in the dairy industry and have demonstrated benefits in both milk production and composition, including greater fat yields compared to corn grain (CG) [2]. However, knowledge is still limited regarding its effects on methane (CH_4_) emissions and animal performance when used as a highly degradable fiber-based energy source. Further research is needed to optimize its inclusion and better understand its impact on the sustainability and efficiency of dairy production systems.

Several studies have previously investigated the replacement of CG with SH at different inclusion levels. For example, Ipharraguerre et al. [3] reported that partial substitution up to 30–40% of dietary dry matter (DM) supported similar DMI, nutrient digestibility, and milk yield, although higher inclusion levels reduced non-structural carbohydrate digestion. Similarly, the review by Ipharraguerre and Clark [4] highlighted that SH can replace ~30% of dietary DM as grain in high-concentrate diets or ~25% when replacing forage, provided that effective fiber is maintained. Ranathunga et al. [5] also observed that replacing starch with SH and distillers grains reduced dietary starch content while sustaining milk production.

Dry matter intake in dairy cows is regulated by both physiological mechanisms and rumen fill, with the relative importance of each varying throughout the stages of lactation [6]. Evidence suggests that even minor changes in dietary starch levels can significantly affect DMI and milk production in early lactation cows [7]. These effects are explained through the hepatic oxidation theory, which, according to Allen et al. [6], plays a predominant role at the onset and toward the end of lactation periods, during which energy utilization patterns shift. Consequently, these changes in intake and energy metabolism could result in variations in CH_4_ emissions per unit of energy-corrected milk (ECM) beyond what would be expected solely from changes in ruminal metabolism.

More recently, Juckem et al. [8] demonstrated that reducing dietary starch concentration from 27% to 21% of DM with replacing CG with SH up to 9% of dietary DM resulted in greater CH_4_ emissions and intensity without impairing milk yield in lactation cows.

Methane generation is affected by the feed ingredients in ruminant rations. According to Chen et al. [9], SH produce a greater amount of CH_4_ per unit of fermented DM compared to other feeds such as CG (52 vs. 42.1 mL/g incubated DM, respectively). This is related to the greater acetate proportion in the volatile fatty acid (VFA) profile of fiber-rich diets, like those supplemented with SH, which promotes the activity of hydrogenotrophic methanogens that use the hydrogen produced during fiber fermentation to generate CH_4_. On the other hand, in a recent in vitro trial, Buraschi et al. [10] compared ryegrass-based diets supplemented with either CG or SH using rumen fluid from ewes previously fed the same diets. The study reported that CH_4_ production per unit of DM (CH_4_/DM) was significantly higher in the CG treatment compared to SH (5.56 vs. 3.7 g/kg DM, *p* = 0.0287), indicating that changes in ruminal microbiota could explain the observed results.

Nitrogen efficiency is another way ruminants impact the environment. Their relatively low efficiency in retaining dietary N in animal products [11] leads to significant N losses in the form of excretions. This can contribute to air, soil, and water pollution. Therefore, an effective mitigation approach of environmental impacts should not only reduce CH_4_ emissions, but also improve the N balance, increasing the efficiency of nutritional strategies in reducing the overall environmental burden. In this context, improving N use efficiency (NUE) in ruminants is of particular interest, as it directly affects both animal productivity and environmental sustainability. According to Cantalapiedra-Hijar et al. [12], dietary interventions that optimize the synchronization between energy and N supply can enhance microbial protein synthesis and reduce N excretion, particularly urinary losses of N, which are highly susceptible to volatilization and leaching. This highlights the importance of formulating diets that not only meet the nutritional demands of the animals, but also minimize nutrient losses to the environment. Hence, evaluating alternative feed ingredients and their impact on NUE, alongside CH_4_ mitigation strategies, is crucial for developing more sustainable livestock production systems.

The aim of our study was to evaluate the impacts of partially replacing CG with SH (100% replacement of CG corresponding to ≈7.5% of the total diet) on enteric CH_4_ production, N and energy use efficiency, milk production and composition, the ruminal environment, and the microbiome of lactating dairy cows. By targeting the replacement of starch (CG) with highly degradable fiber (SH), this study further addresses knowledge gaps on how SH inclusion influences nutrient efficiency and environmental outcomes in dairy systems, including its implications for nitrogen (N) efficiency, energy partitioning, and the ruminal microbiome under different replacement levels.

## 2. Materials and Methods

### 2.1. Experimental Site

Our study was conducted from September to December 2021 at the Methane Laboratory of the National Institute of Agricultural Technology, Balcarce Experimental Research Station (EEA INTA Balcarce), Argentina (37°45′37″ S, 58°18′02″ W, and 131 m above sea level). The climate is temperate, with a mean annual rainfall of 795 mm and 14 °C mean annual temperature (INTA-Balcarce weather station, 1961–2018). The experiment was approved by the Institutional Committee of Care and Use of Experimental Animals (CICUAE INTA-CeRBAS) under protocol number 219/21, approved on 13 April 2021.

### 2.2. Animals and Treatments

Six multiparous, lactating, Holstein cows from the experimental herd of the dairy unit of the EEA INTA Balcarce were selected and transferred to the Methane Laboratory at 40 ± 10 days in milk (DIM). Our study used a replicated 2 × 2 Latin square design with three replicates, whereby six cows were allocated one of two dietary treatments (*n* = 3 per treatment). Each experimental period lasted for 25 days, with 17 days of acclimatization and 8 days of measurement. Cows were assigned to treatment CG or SH in pairs, defined by calving date, milk production (22.6 ± 5.24 L), number of lactations (1.8 ± 3.17), body condition score (3.5 ± 0.45), and body weight (555 ± 54.5 kg). The dietary treatments were isonitrogenous (20.4% crude protein, CP) consisting of a 50:50 (DM basis) forage to concentrate ratio. Basal forage consisted of whole-plant corn silage in both experimental groups. The dietary treatments were defined by the type of energy source in the concentrate. In the soybean hulls (SH) diet, soybean hulls partially replaced corn grain, resulting in 23.6% SH versus 16% SH in the control diet (CG), equivalent to ≈7.5% of the total diet DM as replacement. Their composition is described in Table 1. Both concentrates were pelleted, which minimized particle size differences and likely reduced their contribution to physically effective NDF.

Additional cows were initially considered to increase replication; however, they could not complete the study due to health and adaptation issues related to respiration chamber housing. Therefore, a replicated 2 × 2 Latin square was chosen, as it maximizes statistical power with the number of cows available while balancing feasibility and animal welfare considerations.

### 2.3. Measurements

#### 2.3.1. Animal Performance and Dry Matter Intake

Cows were milked twice daily using a portable, single-cow, milking machine during the whole experiment. The same machine was used both in the acclimatization pens and inside the respiration chambers. It was equipped with a 25 L milk tank, allowing for the milk to be collected, weighed using a digital scale (kg), and sampled individually. Fat-corrected milk (FCM) was calculated using Equation (1) and energy-corrected milk (ECM) using Equation (2), as suggested by a previous study [13]:

FCM = 0.4 × milk production (kg/d) + 15 × milk fat (kg/d)(1)

ECM = 12.95 × milk fat (kg/d) + 7.2 × milk protein (kg/d) + 0.327 × milk production (kg/d)(2)

From day 18 to 25 of each experimental period, milk samples from each milking shift (morning and afternoon) were collected and proportionally weighted to represent each milking shift in a composite sample per cow per day. The milk samples collected from day 18 to 21 were used for N determinations, while the samples from day 22 to 25 were used to determine milk composition and fatty acid profile. The samples were collected in sterile 100 mL containers, refrigerated without preservative and sent immediately to the Milk and Dairy Products Quality and Technology Laboratory of EEA INTA Balcarce, for composition analysis (fat concentration, total solids concentration, non-fat solids concentration, proteins, and lactose) using the spectrophotometric (IR) method (Foss 605B Milko-Scan, Foss Electric, Hillerød, Denmark). Fatty acid profile in milk was determined by gas chromatography with the Ce-1j-07 method [14].

Feed was offered twice daily to ensure ad libitum intake. During the last 8 days of each period, dry matter intake (DMI; kg DM/d) was measured by the gravimetric difference in offered and refused feed (DM basis). For this, samples of approximately 500 g were collected daily from the offered silage and concentrates, and a composite sample was made per ingredient per period. Every day, refused feed was collected, weighed, and sampled before the morning milking. The refusals were combined per period for each cow and proportionally to the refusal per day. Two types of composite samples were collected, one for N balance that included the 4 days in the pen before entering the chambers (from 18 to 21 days), and another one for the analysis of chemical composition during the last 3 days where the cows were inside the chambers (from days 22 to 25).

All samples of offered and refused feed were dried at 60 °C in a circulated-air oven until constant weight to determine the DM concentration. Then, samples were processed in a Willey mill with a 1 mm mesh and analyzed at the feed quality lab (INTA Rafaela, Rafaela, Argentina) to determine the concentration of analytical DM at 105 °C for 24 h, ash [15], organic matter (OM) as the difference between DM and ash concentration, total N (TN, g/d and %) [16], crude protein (CP = TN × 6.25, kg/d and %), neutral detergent fiber (NDF), and acid detergent fiber (ADF, kg/d and %) according to Van Soest et al. [17], and were adapted for the Ankom 200 Fiber Analyzer (Ankom Technology, Fairport, NY, USA) based on ISO 13906:2008, lignin (ADL, kg/d and %) [18], ether extract (EE, kg/d and %) [15], starch (kg/d and %) [19], and indigestible NDF (NDFi, kg/d and %, remaining after 12 days of in situ incubation) [20]. NDF, GE, and NDFi were also analyzed in feces to use as internal markers to estimate total fecal output. Apparent DM digestibility (DMD) was calculated as the difference between DMI and total fecal output using the equation DMD = (DMI − total fecal output)/DMI × 100. Digestibility of NDF and GE was calculated using the same equation based on the intake of DNF or GE and their respective proportions in feces [21].

#### 2.3.2. Enteric Methane Production

The Methane laboratory of INTA Balcarce is equipped with 2 open-circuit respiration chambers (RCs) to measure enteric CH_4_ emissions and 12 individual training pens.

##### Respiration Chamber Description and Operation

The structure of the RC (walls, doors, and ceiling) is made of double stainless steel (AISI 304) panels filled with polyurethane and double-glazed two-sided windows of 3 m^2^. Each RC has a volume of 23 m^3^ and are built next to each other, sharing the wall and window in the middle, allowing for the animals to see each other and the surroundings. The RC has two swing doors: the rear one for the entry and exit of the animals and the front door for personnel use. Both RC have an automatic emergency opening, with a magnetic lock and weight such that it opens and allows for fresh air to enter the RC in case of a technical failure. Each RC has a stainless steel crate and feeder inside, mounted on a concrete floor. A rubber carpet is placed on the section inside the crate. A drainage at the rear and outside the crate is provided with a float valve to fill the drainage with water. After cleaning the RC a pipe trap is opened from outside the RC to clean the drainage and closed again until the next cleaning time.

Each RC is equipped with an air conditioning unit for temperature and relative humidity control. A regenerative turbine (2RB 420-7HA31, 150 m^3^/h, Greenco Industry Co., Ltd., Taizhou, China) outside each RC extracts the air, generating a slight negative pressure. Fresh air enters through a polyvinyl chloride (PVC) tube to the RC recirculated air duct at the upper front and leaves from the rear edges of the RC, opposite to the air inlet. The airflow is controlled by a manual valve connected to the outlet pipe of the system. Airflow is measured with differential pressure at a straight section of PVC duct of 25 mm i.d. and 1 m length that guarantees a laminar flow. Outlet air is then conducted outside the building through PVC tubing. To achieve adequate ventilation, a fan is placed inside each RC. The air inlet is sampled from a common tube to both RCs, and the outlet air is sampled independently from each RC after the airflow measurement section. These sample lines are connected to a continuous sampling diaphragm vacuum pump (Bühler-Technologies, Ratingen, Germany). This pump brings the sampled air from both RCs and fresh air to a sample switching device (Adox, Buenos Aires, Argentina) that sends the sample from each RC outlet and the fresh inlet air sequentially to a Peltier sample cooler (PKE 512, Bühler-Technologies, Ratingen, Germany) set at 4.5 °C dew point. Gas concentrations are then measured using a gas analyzer with O_2_ (paramagnetic), CO_2_ (infrared IR 1520), and CH_4_ (infrared GFX) detectors (Servopro 4100, Servomex, East Sussex, UK).

Each RC works independently from the other. A bespoke software controls the operation, control, and supervision of the entire system, data collection, and storage. The system continuously measures the temperature, humidity, pressure, and outlet airflow individually in each RC. Once the concentration of gases is recorded, the system also records all the parameters measured continuously. Video cameras inside each RC allow for monitoring of animals’ behavior. All real-time data and videos are accessible remotely.

Although the sampling pump runs continuously, the gas switching device allows for starting with air samples from RC1, then RC2, and finally fresh air, and then the cycle starts again. The length of time that the sample is being pumped to the analyzer from each sampling point is manually selected according to the time it takes the sampling line and analyzer to clean the remains of the sample collected before and start measuring the sample of the next RC.

##### Calculation of Methane Emissions

Each RC is switched on at least 12 h before the measurement period begins to maintain the air inside the RC at the given temperature, humidity, and pressure, airflow, and get the gas analyzer warmed up and fully functioning. Doors are opened for feeding and enter the animal on its first day. Once the animal is inside the RC, the doors are closed and will be opened again for the next feeding/milking time. Every time the doors are opened and closed, or any unexpected failure occurs, the time is registered to examine in the database and correct accordingly. These gaps are completed by the mean value of four data points registered before and after the period that needs correction.

The protocol to calculate the CH_4_ emission in L or g per day follows the recommendation described by Pinares-Patiño et al. [22]. Briefly, the gas (CH_4_ or CO_2_) concentration measured inside the RC minus its concentration in the fresh air is multiplied by the dry ventilation rate (DVR) corrected by the standard temperature and pressure (DSTPVR) and then corrected by the gas recovery rate of each chamber and the calibration of the gas analyzer. For a given time point, the CH_4_ emission is calculated using the following set of equations suggested by [22]:

CH_4_ emission (L/min) = (DSTPVR × ([CH_4_ ppm]/1,000,000))/gas recovery rate.

DSTPVR (L/min) = [(Air pressure × DVR)/(Chamber T + 273.15)] × 273.15/1013.25, where pressure is in hPa, Dry gas VR is in L/min, Chamber T is the chamber temperature in °C.

DVR (L/min) = Wet VR × [(100 − VMR)/100], where Wet VR is the ventilation rate recorded from the flow meters (L/min), VMR is the Volume Mixing Ratio of moisture (%).

VMR (%) = 100 × PWP/air pressure, where PWP is the partial water pressure (hPa), and the air pressure in hPa.

PWP (hPa) = (6.1117675 + 0.4439 T + 0.014305 T^2^ + 0.000265 T^3^ + 0.00000302 T^4^ + 0.0000000204 T^5^ + 0.00000000006388 T^6^) × RH/100, where T is chamber temperature (°C) and RH is the chamber relative humidity (%).

Daily emissions are converted from L/day to g/day using the conversion: 1 g CH_4_ = 1.3962 L CH_4_. The final value of CH_4_ is corrected by the time sequence utilized (e.g., 6 min). Finally, the CH_4_ emission per day for each chamber is calculated by the addition of each value within each 24 h period.

##### Calibration and Recovery Rates

The calibration of the gas analyzer is run manually at the beginning and the end of each experimental period. To do this, ultrapure N_2_ gas is used to calibrate the analyzer to zero. Certified span gases in a mix with N_2_ are specifically bought for each experiment to manually calibrate each sensor individually (CO_2_ at 0–5000 ppm, CH_4_ at 0–800 ppm, and O_2_ at 0–21%). Deviation from the expected concentration in the analyzer is corrected before starting a new period, or we back-calculate the measured data if detected at the end of each period.

Tests to determine the recovery rates were performed with ultrapure (99,99%) CO_2_. A flow control rotameter was set at 2 L/min to inject pure CO_2_ until a plateau was reached and recorded for 1 h. The average of measured CO_2_ in L/min was calculated only during the plateau phase. To calculate the recovery rate, the mean measured emission of CO_2_ (L/min) is multiplied by 100 and divided by the injected CO_2_ in L/min. The percentage obtained represents the gas recovery rate. In our system, after seven recovery rate measurements on each chamber, an average (±standard deviation) of 99.3 ± 6.68 and 104.6 ± 6.20% recovery rate were observed for RC1 and RC2, respectively.

##### Respiration Chamber Measurement Protocol

In this study, each experimental period comprised 17 days of acclimatization to the daily routine and diets, followed by 8 days of measurement. Starting on day 3 of the acclimatization period, cows began their adaptation to the respiration chambers, staying in the chambers for 1 h with the doors open and gradually extending their stay inside to acclimatize to the new environment. Cows entered chambers in pairs, 1 to each chamber, on day 22 of each period after the morning milking, and remained over 3 days with free access to feed and water.

The first measurement of each cow was made at 70 ± 5 DIM. At the end of the first period, cows returned to the same individual training pen and began the second experimental period with the opposite dietary treatment for 17 days, followed by the second CH_4_ measurement at 95 ± 4 DIM.

Each chamber was set to maintain a constant air flow at 140 m^3^/h, between 15 and 18 °C, between 60 and 80% humidity, and air pressure slightly negative (mean −26.5 Pa). The time sequence between recordings was set at 6 min intervals, allowing for 2 min to inject a sample from each sample point.

Each day, the chambers were opened for 30 min, one at a time, twice a day for cleaning, feeding, and milking, while both the rear and front doors remained fully open to ventilate the air inside the chamber.

#### 2.3.3. Energy Balance and Energy Use Efficiency

The energy balance (EB) for each dietary treatment was calculated as the difference between the energy consumed and the energy used for maintenance and production using the following equation:

EB = Metabolizable Energy (ME, MJ/d) − 0.08 × BW^0.75^ (body weight, kg) – E_Milk_

The Energy Use Efficiency (EUE) was determined as the proportion of energy consumed that was used for production using the following equation:

EUE = E_Milk_/GEI.

The DE was calculated as the difference between the GEI and the energy excreted in the feces (DE = GE − E_feces_) [23]. The ME was calculated as the absorbed energy plus the fermented energy minus the energy lost in the urine (ME = Absorbed energy + Fermented energy − E_urine_). The absorbed energy represents the energy contained in milk production (E_milk_), and the energy retained in body weight and body condition score changes. The E_milk_ was estimated as E_milk_ = kg of fat in milk × 0.03932 MJ/kg of fat + kg of protein in milk × 0.02384 MJ/kg of protein + kg of lactose in milk × 0.01673 MJ/kg of lactose. Body weight (BW, kg) was measured with an electronic scale and body condition score (BCS) was registered by a trained person and was assessed on a 1 (emaciated) to 5 (obese) scale, with 0.25 increments, according to Edmonson et al. [24]. Both were measured on two consecutive days at the beginning (day 1) and at the end (day 25) of each experimental period. The fermented energy corresponds to the energy released as methane (E_methane_): E_methane_ = CH_4_ production (g/d) × 0.0556 MJ/g (World Nuclear Association, n.d.). The E_urine_ was estimated according to Van Soest [23] as 4% of GEI (E_urine_ = GEI (MJ/d) × 4/100).

#### 2.3.4. Nitrogen Balance and Nitrogen Use Efficiency

The NUE was calculated as the ratio of average milk N to intake N. To determine the N balance, the N consumed and excreted were quantified. The intake N (g/d) was estimated from the difference between N content in the offered and in the refused feed. The Total N excretion (g/d and % of N intake) was calculated by adding Fecal N and Urinary N. The Productive N (g/d and % of N intake) was calculated as the percentage of Milk N multiplied by the kg of milk produced per day. The retained N (g/d and % of N intake) corresponds to intake N minus Total N excretion, and productive N corresponds to Retained N plus milk N.

To measure Total N excretion, daily fecal samples were collected from each cow at four time points (08:00; 12:00; 15:00; 18:00 hs) from days 18 to 21 of each experimental period. For each day, a composite sample was prepared by combining 75 g from each collection. Samples were dried in a forced-air oven at 60 °C for approximately 72 h (or until constant weight was achieved), ground using a Willey knife mill with a 1 mm sieve, and stored for later N analysis.

Urine samples were collected after each milking during the same four-day period. Approximately 500 mL of urine was collected by manual stimulation of the vulva into polyethylene bags. A 15 mL aliquot was acidified with 60 mL of sulfuric acid (H_2_SO_4_) 0.072 N and stored at −20 °C until analysis for TN by the Kjeldahl method [16]. The remaining volume was refrigerated at 5 °C for daily pooled samples and creatinine determination using the Jaffé colorimetric method [25]. Daily urinary volume was estimated using the creatinine concentration and assuming an excretion rate of 29 mg/kg BW [26].

On day 21 of each period, blood samples were collected from the coccygeal vein. Serum was obtained by centrifugation (2000× *g* for 15 min at 4 °C) to determine glucose (mg/100 mL, Enzymatic blood glucose, Wiener Laboratory, Rosario, Argentina), creatinine (mg/L), and urea (BUN, mg/L, Uremia test kit, Wiener lab.).

Milk samples of 100 mL each were collected from each milking in sterilized bottles during the four sampling days and frozen at −20 °C for subsequent analysis of TN using the Kjeldahl method [27].

#### 2.3.5. Rumen Variables and Microbiome Composition

On day 25, once the animals exited the chambers, ruminal fluid samples (~400 mL) were collected 4 h after feeding using an esophageal tube using a vacuum trap system [28]. Immediately after collection, pH was measured with a calibrated digital pH meter (Cole-Parmer model Digi-sense). Samples were then filtered with a double cheese-cloth and divided into three aliquots with duplicates. One aliquot of 50 mL without preservatives was stored at −80 °C for ruminal microbiome analysis. A second aliquot of 5 mL of ruminal fluid was preserved with 0.05 mL pure H_2_SO_4_ and stored at −20 °C to determine VFAs concentration [29]. And a third aliquot of 4 mL of ruminal fluid was preserved with 4 mL of 0.2 N HCl and stored at −20 °C to determine ammonia nitrogen (NH_3_-N) concentration [30].

For microbiome analysis, DNA was extracted following the protocol described by Yu and Morrison [31], and its quality was verified using 0.8% agarose gel electrophoresis. The V4 region of the bacterial 16S rRNA gene was analyzed by Novogene (Novogene Co, Beijing, China). De-multiplexed amplicon paired-end reads were processed using the DADA2 (Divisive Amplicon Denoising Algorithm) package of the R software [32]. The reads underwent quality checking and filtering, followed by merging overlapping forward and reverse reads (250 bp) to obtain full denoised sequences. Amplicon sequence variants (ASV) were inferred, and the abundance of each ASV across the samples was calculated after removing chimera sequences. Taxonomic assignment of ASV was performed using the SILVA database (version 138.1) [33] for bacterial 16S rRNA gene sequences.

### 2.4. Statistical Analysis

Statistical analyses were performed using the GLIMMIX procedure of SAS version 9.4 [34], which allows for fitting linear mixed models appropriate for the replicated 2 × 2 Latin square design. The experiment comprised three independent Latin squares, each including two cows, two periods, and two treatments (*n* = 3 squares). The cow was defined as the experimental unit (*n* = 6). Within each square, treatments were randomly assigned so that each cow received both treatments across two periods, thus controlling carryover and period effects.

For variables measured outside the respiration chambers (e.g., N intake and excretion), data were analyzed using a linear mixed model including fixed effects of treatment, period, and square. Cows were modeled as a random effect nested within the square to account for variation among animals and repeated measurements across periods.

For variables measured inside the chambers (e.g., methane emissions, dry matter intake, milk production, and milk composition), the model was extended to include chambers nested within cows as a random effect to account for repeated measurements taken within chambers.

For microbiome analysis, variation was applied to account for the varying depths of coverage across samples. Good coverage was calculated to ensure that all samples reached a minimum value of 0.97. Significant effects (*p* ≤ 0.05) on bacterial composition were estimated using permutational multivariate analysis of variance with vegan package [35] in R version 4.2.0 [36] for ‘Period’ (1 and 2) and Treatment (CG and SH). Differences in abundance for bacterial ASVs between treatments were performed by grouping the ASVs at the Phylum, Class, and Genus level and retaining only the most abundant taxa (>5%).

Correlation between variables was tested by the PROC CORR procedure using Pearson correlation coefficients in SAS Studio (SAS; University Edition, SAS Institute Inc., Cary, NC, USA).

Principal Component Analysis (PCA) was performed using the PROC PRINCOMP procedure in SAS Studio to reduce the dimensionality of the dataset and to identify underlying patterns among the variables. Prior to the analysis, all continuous variables were standardized to have a mean of zero and a standard deviation of one, ensuring comparability across different scales. The number of components retained was based on eigenvalues > 1 and the percentage of explained variance.

Results are presented as mean values and standard error of the means. Pairwise comparisons between treatments were performed using Tukey’s test, and statistically significant differences were declared at *p* < 0.05, and a trend was considered when 0.05 ≤ *p* ≤ 0.10.

## 3. Results

### 3.1. Feed Intake and Digestibility

Cows fed the SH diet showed significantly greater DMI and tended to have greater OM intake and GEI than cows fed the CG diet (Table 2). Although diets were formulated to be isonitrogenous, CP intake was different between treatments, being 4% greater for cows fed SH compared to CG (*p* < 0.05). As expected, the intake of fiber fractions (NDF and ADF) was greater, and starch intake was lower in cows fed SH than those fed CG. No differences were observed on intake of EE or ADL between dietary treatments (*p* > 0.05).

Cows fed the CG diet showed significantly greater DMD (*p* < 0.001), gross energy digestibility (GED, *p* = 0.004), and neutral detergent fiber digestibility (NDFD, *p* = 0.037) compared to those fed SH (Table 2).

### 3.2. Milk Production and Fatty Acid Profile

No significant differences were found between dietary treatments in total milk, FCM, or ECM production (Table 3). Furthermore, milk composition was also not different between treatments. Regarding feed efficiency, greater efficiencies were observed in cows offered the CG treatment compared to the SH cows, showing statistically significant differences when measured as milk/DMI (*p* = 0.003), FCM/DMI (*p* < 0.001), and ECM/DMI (*p* < 0.001).

Dietary treatments significantly influenced selected milk fatty acids (Table 4). The 15:0 iso (*p* = 0.031), 15:0 anteiso (*p* = 0.012), and 17:0 anteiso (*p* = 0.001) proportions were greater in milk from the SH cows compared to the CG cows. There was a tendency (*p* = 0.064) for the proportion of 13:0 fatty acids to be greater in milk from CG cows than from SH cows, while the alfa-linolenic acid 9c, 12c, 15c-18:3 (n-3) tended (*p* = 0.077) to be greater in milk from SH cows than CG cows. No other measured fatty acids showed significant treatment effects (*p* > 0.05).

### 3.3. Enteric Methane Production

Daily CH_4_ production tended (*p* = 0.075) to be greater in cows fed a SH diet (Table 5). The DMI measured inside the chambers (DMI chambers) was significantly greater (*p* = 0.03) for cows consuming the SH rather than the CG diets. The proportion of the GEI and DEI lost as enteric CH_4_ were significantly greater (*p* = 0.018 and *p* = 0.017) for cows fed SH than those offered CG. No significant differences were found on CH_4_ yield (g/kg DMI chambers; *p* = 0.345) or intensity (g/kg milk) of cows fed either the SH or CG diet (*p* = 0.387) (Table 5).

### 3.4. Balance and Efficiency of Energy and Nitrogen Use

Nitrogen and energy balance data are presented in Table 6. Intake N (g/d) was not different between treatments, with a significant difference in fecal N excretion (*p* < 0.001), which was greater in the SH treatment. In line with this result, total N excretion was significantly greater in this treatment (*p* < 0.001), while milk N was significantly greater in the CG treatment (*p* = 0.002).

Nitrogen use efficiency was not affected by treatment, but N balance tended to be greater in the CG treatment (*p* = 0.056). Digestibility of GE, DE, and ME did not show significant differences between treatments.

The dietary treatments significantly influenced energy utilization without affecting the EB (*p* = 0.515). Cows fed CG presented significantly lower energy loss through feces in (*p* = 0.002), urine (*p* = 0.015), and methane emissions (*p* = 0.023) compared to the SH group. However, when energy loss was expressed relative to GE intake, no significant difference (*p* = 0.191) was observed between treatments. The CG treatment promoted more efficient energy utilization through milk synthesis, with both milk energy output (*p* = 0.009) and the proportion of GEI partitioned to milk (*p* < 0.001) being significantly greater than in the SH treatment.

Blood metabolite analysis showed no significant effect on glucose, creatinine, and BUN concentrations (*p* > 0.05; Table 6).

### 3.5. Rumen Variables and Microbiome Composition

Neither ruminal fluid pH (*p* = 0.542) nor NH_3_-N concentration differed between treatments (*p* = 0.673; Table 7). Analysis of total and individual VFA concentration, molar proportion, and acetate to propionate ratio (A:*p*) showed no treatment effects (*p* > 0.05; Table 7).

The microbiome analysis showed that a total of 2241 ASV were identified from ruminal samples. Twenty-one taxonomic assignments were detected at the Phylum level (relative abundance (RA) > 0.5%); Firmicutes, Euryarchaeota, Proteobacteria, and Bacteroidota were the most abundant, but none of these were affected by diet (Table 8). Firmicutes and Bacteroidota accounted for 82.7 and 85.6% RA in cows offered the SH and CG dietary treatments, respectively (Table 8). Rumen Cluster C abundance was not affected by treatment, and overall values remained below 0.01%. Ruminal microbiome differed at the Phylum level for the RA of Patescibacteria, which tended to be greater in CG cows than SH cows (*p* = 0.092), and Nitrospirota, which was significantly greater in SH cows than CG cows. The preliminary analysis of genera over 0.1% RA identified 167 different genera, although only 20 were considered dominant over 0.5% RA (Table 9). *Ruminococcus* (28.84 vs. 30.27%), *Prevotella* (7.92 vs. 7.89%), and *Christensenellaceae* R-7 group (3.17 vs. 4.51%) were the most abundant, but no effect from diet was detected for these taxa (*p* > 0.05). Only one genus from the Saccharimonadaceae family, *Candidatus Saccharimonas*, tended to be greater in SH cows than CG cows (*p* = 0.095; Table 9).

**Figure 1:**
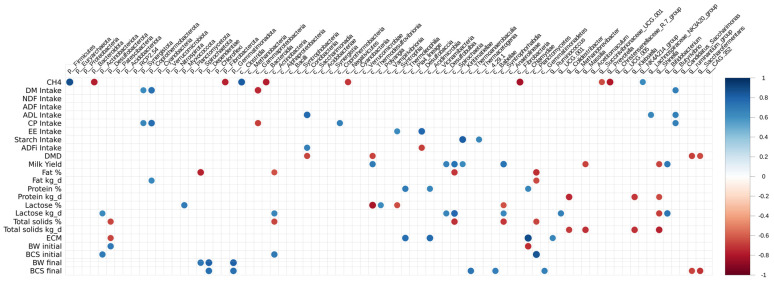
Correlation between the different genera of ruminal fluid microorganisms across the dietary treatments of Corn Grain (CG) or Soybean Hulls (SH) and the most relevant productive variables in this study.

Significant correlations were detected between some dominant taxa from ruminal microbiome and the main animal productivity variables such as CH_4_ production, DMI, and ECM (Figure 1). Methane production was associated with the UCG001 (r = 0.598; *p* = 0.067) and NK4A214_group (r = 0.638; *p* = 0.049) from the Ruminococcaceae family. Methane was negatively correlated with *Fibrobacter* (r = −0.824; *p* = 0.003) from Fibrobacterota and positively correlated with *Succinivibrio* (r = 0.758; *p* = 0.010) from Succinivibrionaceae family. From phylum Bacteroidota, the genus *Prevotella* exhibited a strong negative correlation (r = −0.79; *p* = 0.005) with CH_4_ production. On the other hand, DMI was positively associated with genus *Butyrivibrio* (r = 0.673; *p* = 0.032) and *Lachnospiraceae* XPB1014 (r = 0.676; *p* = 0.032) and tended to correlate with *Lachnospiraceae* UCG 002 (r = 0.586; *p* = 0.074) and Methanotermobacter (0.5508; *p* = 0.098). ECM was positively correlated with *Ruminococcus* (r = 0.658; *p* = 0.038) and *Lachnospiraceae* 010 (r = 0.654; *p* = 0.040) and negatively correlated with *Methanobrevibacter* (r = −0.571; *p* = 0.084), *Methanosphaera* (r = 0.650; *p* = 0.041), *Chitinophaga* (r = −0.582; *p* = 0.077), and *Eubacterium ruminantium* (r = 0.619; *p* = 0.056) (Figure 1). We also analyzed correlations with the α-linoleic acid, which tended to be greater in milk from SH cows than CG cows, and significant positive associations were found with *Fibrobacter* (r = 0.716; *p* = 0.0196), *Succinivibrio* (r = 0.761; *p* = 0.010), and *Prevotellaceae*_UCG001 (r = 0.694; *p* = 0.025).

## 4. Discussion

Cows fed the SH diet tended to have greater enteric CH_4_ emissions and had significantly greater Ym, which is primarily attributed to the greater DMI recorded in this treatment, compared to cows fed CG. These results are in agreement with a previous study [37] evaluating the inclusion of SH to replace CG in a TMR using a forage-to-concentrate ratio of 60:40 (DM basis). These authors reported significantly greater DMI of cows fed a diet containing 50% SH of the concentrate part of the diet, with a concomitant increase in CH_4_ production (g/d) as a result of greater supply of fermentable substrates. Although SH promoted greater DMI, it did so at the cost of increased enteric CH_4_.

When compared to previous studies, diverse factors may explain the lack of expected effect of increasing the proportion of SH in our experiment. Firstly, a previous report [10] evaluated the dietary treatments with sheep’s ruminal fluid, in which the microbiome can differ from that of cattle due to host-specific factors such as differences in rumen size, digesta passage rate, feed intake behavior, and immune modulation [38]. Secondly, the proportion of SH inclusion in the diets of our study (7.5% of SH in the diet) differ from the one used in vitro (100% SH substrate) [4]. Thirdly, it is also worth noting that, in our study, a reduction of 7.5% on the inclusion of CG and an increment of 7.5% on the inclusion of SH from the CG- to the SH-based diets resulted in only 4% more NDF, 3.2% more ADF, and 2.6% less starch. As no differences were detected in ruminal fermentation parameters (pH, VFA, acetate:propionate ratio, NH_3_-N) between dietary treatments, this suggests that the difference in the chemical composition between the treatments, reflecting a change in the energy source, was not enough to significantly modify the ruminal environment and hence make changes in the microbial population. This stability in ruminal fermentation characteristics may be partially explained by the resilience of the core ruminal microbiome [38], which helps maintain fermentation function despite dietary changes. The microbial community was more diverse in SH cows than CG cows, and certain taxa, such as *Candidatus saccharimonas* and *Patescibacteria*, were more abundant, although their role in CH_4_ production remains to be clarified.

Principal Component Analysis revealed that the PC1 accounted for 48.27% of the total variance analyzed in this study and was primarily driven by variables related to DMI, NDF, ADF, and CH_4_ production. This suggests that dietary intake and fiber concentration are the primary sources of variation in the dataset, which aligns with existing knowledge regarding their influence on ruminal fermentation and CH_4_ emissions. The positive correlations observed between DMI, NDF, ADF, and CH_4_ production are consistent with previous findings [39], reinforcing the role of fiber intake in driving enteric CH_4_ emissions.

At the microbial level, CH_4_ production tended to be greater in cows offered the SH diet, and the main methanogenic archaeon, the genus *Methanobrevibacter*, was more abundant in SH cows, although not significantly different from CG cows. This observation aligns with Henderson et al. [38], who reported that *Methanobrevibacter* is consistently the dominant archaeon in the rumen across diverse ruminant species and diets, but its relative abundance may not vary significantly in response to dietary changes. The genus *Ruminococcaceae* NK4A214, known for its involvement in fiber degradation and linked to biohydrogenation processes [40], was, in our study, strongly associated with CH_4_ production in both diets. Despite this, no direct correlation was found between *Ruminococcaceae* abundance and NDF digestibility or milk fatty acid profile, suggesting a complex interaction between microbial structure and function in CH_4_ dynamics.

Regarding diet digestibility and intake, our results indicate that DMI was significantly greater in cows in the SH treatment compared to those in the CG treatment. Despite greater DMI and NDFI in the SH treatment, the GEI did not differ between dietary treatments, suggesting that the increased intake in SH was offset by its significantly lower DMD and GED, consistent with its fibrous nature [3], which may have also contributed to greater CH_4_ emissions.

Despite the lower digestibility, both energy sources (CG and SH) sustained similar levels of milk production and composition. These results suggest that cows on the SH diet compensated for lower energy availability through increased intake, supporting the concept of a compensatory intake strategy. This aligns with the findings of a previous study [41], which proposed that hunger signals increase with greater energy deficits. The lack of differences in milk yield and components further supports the idea that GEI, rather than digestibility alone, drives productive performance under these dietary conditions [42].

Milk fatty acid composition also differed among cows receiving the different dietary treatments, with greater concentrations of branched-chain fatty acids in SH, as expected, due to increased fiber intake and associated microbial activity. However, these differences did not correlate with CH_4_ emissions. Polyunsaturated fatty acids were more abundant in milk from SH-fed cows, possibly due to a shorter rumen retention time limiting biohydrogenation [43,44]. This could indicate potential changes in microbial function more than shifts in CH_4_ dynamics.

Regarding NUE, cows fed SH had greater total N excretion as a result of greater fecal and urinary N losses. This reflects reduced synchronization between energy and N availability, typical of fiber-based diets [12]. Milk N was greater in CG cows, indicating more efficient N partitioning than in SH cows. While NH_3_-N and BUN were not affected by treatment, these findings have important implications for N_2_O emissions and environmental impact, complementing the CH_4_-related findings.

In summary, CH_4_ emissions were strongly influenced by DMI and fiber intake, both greater in cows offered the SH diet than those offered the CG diet. The lower digestibility of the SH diet contributed to increased CH_4_ emissions, despite the absence of major shifts in ruminal fermentation parameters or microbial composition. These findings highlight the importance of considering intake dynamics and fiber characteristics when evaluating dietary strategies for methane mitigation in dairy systems.

## 5. Conclusions

Our findings indicate that the inclusion of SH at ≈7.5% of the total diet DM (23.6% in the SH diet vs. 16% in the CG diet) as a partial replacement for CG did not reduce enteric CH_4_ production, without major changes in the dominant taxa and key methanogens of the ruminal microbiome. Although greater levels of intake were observed in cows fed SH compared with cows fed CG, these were not associated with greater milk production, and, rather, a detrimental effect was observed on the efficiency of the use of nutrients.

From an environmental perspective, while SH offers a potential strategy to reduce grain use in dairy cow diets and to achieve highly productive performance when intake is not limited, our results suggest that inclusion at ≈7.5% of total diet DM may lead to greater enteric CH_4_ and N excretion. Therefore, the inclusion of SH as a partial substitute for CG should be carefully balanced, considering its impact on intake-driven emissions. Further research should explore the optimal level of inclusion and dietary adjustments—such as synchronizing N and fermentable energy supply—to improve environmental outcomes while maintaining production efficiency.

## Figures and Tables

**Table 1:** Composition of the total mixed ration of dietary and concentrate treatments, Corn Grain (CG), or Soybean Hulls (SH).

	Diet		Treatment Concentrate	
Components, %	CG	SH	CG	SH
Corn grain	12.50	5.00	25.00	10.00
Soybean hulls	16.00	23.58	32.00	47.15
Corn silage	50.00	50.00	-	-
Soybean expeller	19.30	19.30	38.60	38.60
Urea	0.75	0.67	1.50	1.35
Calcium carbonate	0.57	0.57	1.14	1.14
Magnesium sulfate	0.28	0.28	0.56	0.56
Salt ^1^	0.60	0.60	1.20	1.20
Nutrient composition, % of DM				
DM (% of FM)	67.00	66.90	90.00	89.90
OM	94.05	93.65	92.41	91.62
CP	20.43	20.38	32.50	32.40
NDF	30.48	34.43	28.84	36.74
ADF	21.94	18.70	20.15	26.64
ADL	1.27	1.40	1.21	1.48
Starch	22.62	20.04	11.25	6.09
Ether extract	2.58	2.27	2.21	1.58
NDFi	8.95	9.69	4.68	6.16
Gross energy (MJ/kg of DM)	16.42	16.01	16.15	15.88

DM: Dry matter; FM: Fresh matter; OM: Organic matter; CP: Crude protein; NDF: Neutral detergent fiber; ADF: Acid detergent fiber; ADL: Lignin; NDFi: Indigestible neutral detergent fiber. ^1^ Includes 0.1% of a vitamin and mineral nucleo per kg of nucleo—Manganese 25,000 mg; Zinc 35,000 mg; Copper 10,000 mg; Iodine 390 mg; Selenium 200 mg; Cobalt 200 mg; Vitamin A 2,000,000 IU; Vitamin D3 400,000 IU; Vitamin E 15,000 IU; Monensin 17,500 mg.

**Table 2:** Intake and apparent total tract digestibility of nutrients in lactating cows fed diets supplemented with Corn Grain (CG) or Soybean Hulls (SH).

	Treatment		SEM	*p*-Value
	CG	SH		
Intake, kg/d				
DM	26.41	27.74	1.17	0.034
OM	24.86	25.93	1.05	0.080
NDF	8.01	9.58	0.37	<0.001
ADF	5.00	6.18	0.23	<0.001
ADL	0.35	0.37	0.01	0.164
CP	5.80	6.03	0.20	0.015
Starch	5.73	5.34	0.30	0.015
EE	0.69	0.62	0.04	0.158
GE (MJ/d)	430.39	449.98	18.44	0.060
Digestibility, %				
DM	67.70	63.29	1.37	<0.001
GE	62.82	58.61	1.76	0.004
NDF	35.54	32.43	5.40	0.037

DM: Dry matter; OM: Organic matter; GE: Gross energy; NDF: Neutral detergent fiber; ADF: Acid detergent fiber; ADL: Acid detergent lignin CP: Crude protein; EE: Ether extract; SEM: Standard error of the mean. Values are presented as mean ± SEM. Statistical significance was declared at *p* ≤ 0.05, and a tendency was considered when 0.05 < *p* ≤ 0.10.

**Table 3:** Milk production and milk composition of lactating cows fed diets supplemented with Corn Grain (CG) or Soybean Hulls (SH).

	Treatment		SEM	*p*-Value
	CG	SH		
Yield (kg/d)				
Milk	39.82	39.51	2.98	0.557
4% FCM ^1^	35.87	36.70	0.74	0.506
ECM ^2^	39.57	40.55	0.67	0.401
Fat	1.35	1.34	0.09	0.786
Protein	1.31	1.31	0.07	0.941
Lactose	2.01	2.01	0.13	0.645
Total solids	4.95	4.93	0.30	0.697
Composition (%)				
Fat	3.41	3.42	0.08	0.928
Protein	3.34	3.33	0.10	0.583
Lactose	5.09	5.09	0.09	0.523
Total solids	12.53	12.51	0.25	0.866
Feed efficiency (kg/kg)				
Milk/DMI	1.55	1.40	0.07	0.003
FCM/DMI	1.42	1.25	0.05	<0.001
ECM/DMI	1.57	1.39	0.05	<0.001

FCM: 4% fat-corrected milk; ECM: Energy-corrected milk; DMI: Dry matter intake: SEM: Standard error of the mean. ^1^ FCM = 0.4 × milk yield (kg/d) + 15 × fat yield (kg/d). ^2^ ECM = 0.327 × milk yield (kg/d) + 12.95 × fat yield (kg/d) + 7.2 × protein yield (kg/d). Values are presented as mean ± SEM. Statistical significance was declared at *p* ≤ 0.05, and a tendency was considered when 0.05 < *p* ≤ 0.10.

**Table 4:** Fatty acid profile us proportion in milk of lactating cows fed diets supplemented with Corn Grain (CG) or Soybean Hulls (SH).

Fatty Acid, %	Treatment		SEM	*p*-Value
	CG	SH		
4:0 (butyric)	2.71	2.78	0.052	0.440
5:0 (valeric)	0.03	0.03	0.002	0.116
6:0 (caproic)	1.97	2.05	0.040	0.219
7:0 (enanthic)	0.04	0.03	0.004	0.274
8:0 (caprylic)	1.33	1.36	0.033	0.530
9:0 (pelargonic)	0.06	0.05	0.004	0.416
10:0 (capric)	3.67	3.69	0.137	0.940
12:0 ISO (iso-dodecanoic)	0.29	0.28	0.018	0.674
11:0 (undecanoic)	0.13	0.12	0.010	0.556
12:0 (lauric)	4.59	4.51	0.209	0.780
c9 12:1 (cis-lauroleic)	0.11	0.10	0.007	0.610
13:0 (tridecanoic)	0.18	0.15	0.009	0.064
14:0 (myristic)	13.41	13.27	0.531	0.700
15:0 ISO (iso-pentadecanoic)	0.16	0.21	0.029	0.031
15:0 ANTEISO (anteiso-pentadecanoic)	0.46	0.54	0.020	0.012
c9 14:1(cis-myristoleic)	1.09	1.04	0.056	0.535
15:0 (pentadecanoic)	1.38	1.27	0.061	0.217
16:0 ISO (iso-hexadecanoic)	0.15	0.17	0.032	0.600
16:0 (palmitic)	35.03	34.85	0.809	0.883
c9 16:1 (cis-palmitoleic)	1.52	1.35	0.192	0.249
17:0 ANTEISO (anteiso-margaric)	0.42	0.58	0.040	0.001
17:0 ISO (iso-margaric)	0.44	0.49	0.042	0.092
NI	0.18	0.18	0.013	0.884
17:0 (margaric)	0.53	0.55	0.018	0.574
c9-17:1 (cis-heptadecenoic)	0.13	0.14	0.014	0.814
18:0 (stearic)	6.80	6.57	0.623	0.686
t9 18:1 (trans-elaidic)	0.39	0.36	0.052	0.533
t10-18:1 (trans-10 oleic)	0.73	0.80	0.204	0.780
t11-18:1(trans-vaccenic)	1.43	1.42	0.376	0.982
c9-18:1 (cis-oleic)	15.41	15.29	1.073	0.807
c11-18:1 (cis-11 vaccenic)	0.77	0.74	0.030	0.559
c12-18:1 (cis-12 octadecenoic)	0.26	0.25	0.035	0.516
19:0 (nonadecanoic)	0.07	0.08	0.007	0.354
9t, 12t-18:2 (trans-linoleic acid)	0.15	0.16	0.012	0.677
9c, 12c-18:2 (n-6; cis-linoleic)	2.43	2.38	0.202	0.634
6c, 9c, 12c-18:3 (n-6; cis-γ-linolenic)	0.11	0.11	0.010	0.688
9c, 12c, 15c-18:3 (n-3; cis-α-linolenic)	0.29	0.32	0.014	0.077
9c, 11t-18:2 (conjugated linoleic)	0.46	0.58	0.046	0.197
11c, 14c, 17c-20:3 (n-3; cis-eicosatrienoic)	0.16	0.16	0.011	0.767
5c, 8c, 11c, 14c-20:4 (n-6; cis-arachidonic)	0.19	0.18	0.010	0.420
5c, 8c, 11c, 14c, 17c-20:5 EPA (n-3; cis-eicosapentaenoic)	0.02	0.02	0.001	0.655
24:0 (lignoceric)	0.01	0.02	0.081	0.684
7c, 10c, 13c, 16c-22:4 (n-6; cis-adrenic)	0.03	0.03	0.008	0.053
7c, 10c, 13c, 16c, 19c-22:5 (n-3; cis-DPA)	0.05	0.04	0.005	0.184
Total saturated fatty acids	73.97	73.74	0.843	0.831
Total monounsaturated fatty acids	21.88	21.53	0.570	0.715
Total polyunsaturated fatty acids	3.92	3.98	0.243	0.692
Delta-9 Index	0.246	0.245	0.011	0.837

NI: Not Identified; SEM: Standard error of the mean. The first number (before the colon) indicates the number of carbon atoms in the fatty acid chain. The second number (after the colon) indicates the number of double-bonds in the carbon chain (i.e., whether the acid is saturated or unsaturated). If the number is “0,” it means it is a saturated fatty acid (no double-bonds). If there is a number greater than “0,” it means it is an unsaturated fatty acid (with double-bonds). The letter “c” means the double-bond is in the cis configuration (hydrogen atoms are on the same side of the chain). The letter “t” indicates the double-bond is in the trans configuration (hydrogen atoms are on opposite sides of the chain). The positions of the double-bonds (such as “9c”) indicate at which carbon in the chain the double-bond begins, counting from carbon number 1 of the carboxyl group (-COOH). ISO and ANTEISO indicate types of branching in the carbon chain: ISO means there is a branch at the second-to-last carbon. ANTEISO indicates a branch at the third-to-last carbon. (n-3) or (n-6) indicate the type of essential fatty acid (omega-3 or omega-6), where the first double-bond is at carbon n-3 or n-6 counting from the methyl end (-CH_3_) of the chain. Values are presented as mean ± SEM. Statistical significance was declared at *p* ≤ 0.05, and a tendency was considered when 0.05 < *p* ≤ 0.10.

**Table 5:** Methane production of lactating cows fed diets supplemented with Corn Grain (CG) or Soybean Hulls (SH).

	Treatment		SEM	*p*-Value
	CG	SH		
DMI, ^1^ kg/d	26.41	27.74	1.17	0.030
CH_4_				
g/d ^1^	456.13	484.40	33.24	0.075
g/kg of DMI ^1^	16.80	17.35	1.30	0.345
% of GE intake ^1^	5.69	6.32	0.48	0.018
% of DE intake ^2^	9.14	10.06	0.43	0.017
g/kg of milk ^3^	11.89	12.30	1.47	0.387
g/kg of FCM ^3^	12.85	13.33	0.94	0.538
g/kg of ECM ^3^	11.63	12.08	0.84	0.498
g/kg of milk fat ^3^	327.04	374.44	46.88	0.178
g/kg of milk protein ^3^	335.31	372.28	47.07	0.230

DMI: Dry matter intake; CH: Methane; GE: Gross energy; DE: Digestible energy; FCM: 4% fat-corrected milk; ECM: Energy-corrected milk; SEM: Standard error of the mean. ^1^ DMI was determined for three consecutive days with cows housed in respiration chambers. ^2^ DE = was estimated from energy digestibility measured over the 3 d collection period. ^3^ Milk, FCM, ECM, milk fat, and milk protein yields were measured for three consecutive days (i.e., performance measurements). Values are presented as mean ± SEM. Statistical significance was declared at *p* ≤ 0.05, and a tendency was considered when 0.05 < *p* ≤ 0.10.

**Table 6:** Nitrogen and energy balance in lactating cows fed diets supplemented with Corn Grain (CG) or Soybean Hulls (SH).

	Treatment		SEM	*p*-Value
	CG	SH		
Intake N, g/d	990.33	987.35	27.81	0.905
Fecal N				
g/d	234.86	282.02	7.54	<0.001
% of N intake	24.36	27.85	0.77	0.003
Urinary N				
g/d	383.71	417.14	13.29	0.078
% of N intake	39.43	41.97	1.65	0.245
Total N excretion				
g/d	617.57	700.16	13.92	<0.001
% of N intake	63.91	69.71	1.94	0.028
Milk N				
g/d	203.54	190.45	10.97	0.002
% of N intake	20.47	19.41	0.80	0.120
Retained N				
g/d	357.78	302.18	27.63	0.073
% of N intake	36.08	30.28	1.94	0.028
Productive N ^1^				
g/d	558.05	495.9	37.81	0.064
% of N intake	56.17	50.08	2.73	0.038
NUE	20.47	19.41	0.80	0.120
Intake GE				
MJ/d	420.67	457.99	14.89	0.003
Digestibility (%)	53.92	51.23	3.97	0.191
DE (MJ)	230.45	232.27	16.60	0.781
ME (MJ)	131.86	189.06	27.43	0.134
Energy partition				
E_feces_				
MJ	199.78	226.45	24.26	0.022
% of Intake GE	46.08	48.77	3.97	0.190
E_urine_ ^2^				
MJ	17.16	18.28	0.54	0.015
% of Intake GE	4	4	0.00	1
E_methane_				
MJ	25.26	27.51	1.38	0.023
% of Intake GE	5.73	5.93	0.35	0.377
E_milk_^3^				
MJ	115.74	111.16	5.79	0.009
% of Intake GE	27.25	24.36	0.81	<0.001
Metabolic indicators				
Glucose (mg/100 mL)	40.56	51.16	10.33	0.531
Creatinine (mg/L)	10.28	10.89	0.56	0.443
BUN (mg/100 mL)	56.14	47.17	9.89	0.438
BW (kg)				
Average	586.37	587.00	13.29	0.929
Initial	556.25	558.17	15.38	0.861
Final	594.33	582.17	11.68	0.122
BCS ^4^				
Average	3.20	3.21	0.09	0.905
Initial	3.08	3.13	0.10	0.778
Final	3.21	3.21	0.08	0.999
EB ^5^	74.15	67.24	16.31	0.515

N: Nitrogen; NUE: Nitrogen use efficiency; GE: Gross energy; DE: Digestible energy; ME: Metabolizable energy; E_feces_: Energy excreted in the feces; E_urine_: Energy excreted in the urine; E_methane_: Fermented energy; E_milk_: Energy excreted in the milk; BUN: Blood urea nitrogen; BW: Body weight; BCS: Body condition score; EB: Energy balance. ^1^ Productive N = Retained N + milk N; ^2^ E_urine_: Estimated as the 4% of Intake GE (Van Soest, 1994); ^3^ E_milk_ (MJ) = (kg of fat in milk × 39.32 KJ/kg of fat) + (kg of protein in milk × 23.84 KJ/kg of protein) + (kg of lactose in milk × 16.73 KJ/kg of lactose); ^4^ BSC was assessed on a 1 (emaciated) to 5 (obese) scale, with 0.25 increments, according to Edmonson et al. [26]; ^5^ EB = ME − 0.08 × BW^0.75^ − E_milk_. Values are presented as mean ± SEM. Statistical significance was declared at *p* ≤ 0.05, and a tendency was considered when 0.05 < *p* ≤ 0.10.

**Table 7:** Ruminal fermentation characteristics of ruminal fluid of lactating cows fed diets supplemented with Corn Grain (CG) or Soybean Hulls (SH).

	Treatment		SEM	*p*-Value
	CG	SH		
pH	7.07	7.21	0.18	0.542
NH_3_-N (mg/dL)	12.36	11.20	2.28	0.673
VFA, Mm				
Total	75.33	85.48	11.18	0.452
Acetate (A)	45.02	52.70	7.13	0.359
Propionate (*p*)	19.90	21.28	2.77	0.722
Butyrate	7.53	8.53	1.25	0.356
Isobutyrate	0.49	0.47	0.08	0.874
Valerate	1.18	1.21	0.15	0.848
Isovalerate	0.86	0.89	0.15	0.857
Caproate	0.37	0.40	0.06	0.402
VFA, %				
Acetate (A)	60.12	62.32	1.08	0.110
Propionate (P)	25.44	24.31	1.23	0.389
Butyrate	10.46	9.91	0.66	0.433
Isobutyrate	0.67	0.55	0.06	0.091
Valerate	1.59	1.42	0.10	0.344
Isovalerate	1.23	1.01	0.16	0.331
Caproate	0.51	0.48	0.06	0.747
A:P ratio	2.50	2.63	0.18	0.364

NH_3_-N: Ammonia nitrogen; VFA: Volatile fatty acid; A:P ratio: Molar ratio of acetate to propionate; SEM: Standard error of the mean. Values are presented as mean ± SEM. Statistical significance was declared at *p* ≤ 0.05, and a tendency was considered when 0.05 < *p* ≤ 0.10.

**Table 8:** Ruminal bacterial community at phylum level in dairy cows fed diets supplemented with Corn Grain (CG) or Soybean Hulls (SH).

Phylum	Treatment		*p*-Value
	CG	SH	
RA (%)			
Firmicutes	73.94	70.26	0.217
Euryarchaeota	2.17	3.52	0.730
Proteobacteria	7.17	6.97	0.798
Bacteroidota	11.67	12.43	0.796
Actinobacteriota	1.77	2.86	0.408
Desulfobacterota	0.35	0.34	0.985
Patescibacteria	0.22	0.90	0.093
Acidobacteriota	1.09	1.25	0.560
RCP2-54	0.18	0.29	0.285
Synergistota	0.20	0.11	0.517
Coprothermobacterota	0.13	0.07	0.559
Cyanobacteria	0.55	0.23	0.171
Verrucomicrobiota	0.18	0.28	0.208
Nitrospirota	0.02	0.08	0.045
Myxococcota	0.06	0.08	0.420
Planctomycetota	0.04	0.06	0.375
Spirochaetota	0.03	0.04	0.399
Dependentiae	0.01	0.02	0.295
Chloroflexi	0.03	0.02	0.999
Fibrobacterota	0.01	0.02	0.182
Gemmatimonadota	0.00	0.01	0.177

RA: Relative abundance. Values are presented as mean. Statistical significance was declared at *p* ≤ 0.05 and tendencies at 0.05 < *p* ≤ 0.10.

**Table 9:** Ruminal bacterial community at genus level in dairy cows fed diets supplemented with Corn Grain (CG) or Soybean Hulls (SH).

Genus	Treatment		*p*-Value
	CG	SH	
RA (%)			
Ruminococcus	30.27	28.84	0.845
UCG-001	2.49	2.96	0.596
Colidextribacter	3.03	0.45	0.403
Methanobrevibacter	2.07	2.90	0.710
Massilia	1.07	1.24	0.755
Acetitomaculum	3.59	1.40	0.192
Succinivibrionaceae UCG-001	1.02	0.70	0.675
Prevotella	7.89	7.92	0.994
UCG-005	1.45	0.93	0.111
Christensenellaceae R-7 group	4.51	3.17	0.481
Klebsiella	0.46	0.53	0.789
NK4A214 group	2.83	2.35	0.619
Lachnospiraceae NK3A20 group	1.87	1.56	0.711
Shinella	0.51	0.51	0.998
Bifidobacterium	0.49	0.76	0.648
Butyrivibrio	0.59	0.71	0.689
Candidatus Saccharimonas	0.22	0.79	0.095
[Eubacterium] ruminantium group	0.36	0.71	0.335
Saccharofermentans	0.53	0.67	0.659
CAG-352	0.57	0.48	0.789

RA: Relative abundance. Values are presented as mean. Statistical significance was declared at *p* ≤ 0.05 and tendencies at 0.05 < *p* ≤ 0.10.

## Data Availability

Data supporting reported results can be found upon requirement. Please, contact corresponding author for more information.

## Abbreviations

The following abbreviations are used in this manuscript:

ADF	Acid detergent fiber
ADFi	Indigestible acid detergent fiber
ADL	Acid detergent fiber
ASV	Amplicon sequence variant
BCS	Body condition score
BW	Body weight
CG	Corn grain
CH_4_	Methane
CO_2_	Carbon dioxide
CP	Crude protein
DE	Digestible energy
DIM	Days in milk
DM	Dry matter
DMD	Dry matter digestibility
DMI	Dry matter intake
DNA	Deoxyribonucleic acid
EB	Energy balance
ECH_4_	Energy released as methane
ECM	Energy-corrected milk
EE	Ether extract
EUE	Energy use efficiency
FCM	Fat-corrected milk
GEI	Gross energy intake
IR	Ingestion rate
INTA	Instituto Nacional de Tecnología Agropecuaria
ME	Metabolizable energy
N	Nitrogen
NDF	Neutral detergent fiber
NUE	Nitrogen use efficiency
OM	Organic matter
PCA	Principal component analysis
PVC	Polyvinyl chloride
RA	Relative abundance
RC	Respiration chambers
rRNA	Ribosomal ribonucleic acid
SAS	SAS Studio
SH	Soybean hulls
TN	Total nitrogen
VFA	Volatile fatty acids
